# Electrical cardioversion for early recurrences post pulmonary vein isolation

**DOI:** 10.1007/s10840-022-01368-w

**Published:** 2022-09-09

**Authors:** Gesa von Olshausen, Astrid Paul-Nordin, Jari Tapanainen, Mats Jensen-Urstad, Hamid Bastani, Ott Saluveer, Tara Bourke, Nikola Drca, Göran Kennebäck, Serkan Saygi, Yusuf Turkmen, Per Insulander, Frieder Braunschweig

**Affiliations:** Department of Cardiology, Karolinska University Hospital, Solna, S1:02, 17176 Stockholm, Sweden

**Keywords:** Atrial fibrillation, Catheter ablation, Electrical cardioversion, Early recurrence, Late recurrence

## Abstract

**Background:**

To study the association between timing and success of electrical cardioversion (ECV) for the treatment of early recurrences (ERs) of atrial fibrillation post pulmonary vein isolation (PVI) on long-term rhythm outcome.

**Methods:**

Data of 133 patients ablated for paroxysmal or persistent atrial fibrillation receiving ECV for ERs, i.e., atrial tachyarrhythmia recurrences within 90 days post ablation were analyzed. During 1-year follow-up, patients were screened for late recurrences (LRs), i.e., recurrences after the blanking period.

**Results:**

In 114 patients (85.7%), ECV was successful compared to 19 patients (14.3%) with failed ECV. A higher body mass index (odds ratio (OR) 1.19 (95% CI 1.02–1.39), *p* = 0.029), a lower left ventricular ejection fraction (OR 1.07 (95% CI 0.99–1.15), *p* = 0.079), and performance of ECV > 7 days from ER onset (OR 2.99 (95% CI 1.01–8.87), *p* = 0.048) remained independently associated with ECV failure. During 1-year follow-up, the rate of LR was significantly higher among patients with failed ECV as compared to patients with successful ECV (hazard ratio (HR) 3.00 (95% CI, 1.79–5.03), *p* < 0.001). Patients with ECV performed > 7 days from ER onset had a significantly higher risk of developing LR as compared to patients with ECV performed within ≤ 7 days from ER onset (HR 1.73 (95% CI 1.15–2.62), *p* = 0.009). Performance of ECV > 7 days from ER onset (HR 1.76 (95% CI 1.16–2.67), *p* = 0.008) and failed ECV (HR 3.32 (95% CI 1.96–5.64), *p* < 0.001) remained independently associated with LR.

**Conclusions:**

A failed ECV and performance of ECV > 7 days from ER onset were independently associated with LR.

**Supplementary Information:**

The online version contains supplementary material available at 10.1007/s10840-022-01368-w.

## Introduction

Catheter ablation has become an effective treatment option in patients with symptomatic atrial fibrillation but recurrences of atrial tachyarrhythmias after initially successful catheter ablation are common [1]. Early recurrences (ERs) within 90 days post atrial fibrillation ablation, i.e., during the conventional blanking period, owing to inflammatory changes, healing of the ablation lesions, and changes in neurohumoral status [2, 3] have been shown to be a strong predictor for late recurrences (LRs), i.e., recurrences 3 months post ablation [4, 5]. Optimal management and treatment of ERs are still an unresolved issue. Electrical cardioversion (ECV) is frequently used to restore sinus rhythm in patients with ER. However, its effect on long-term rhythm outcome is not completely clarified. It has been suggested that an aggressive strategy with rapid ECV post ablation and within 24 h from ER onset prevents adverse atrial remodelling and hence decreases the risk of LRs [6]. On the other hand, a “wait-and-see approach” with delayed ECV after recent-onset atrial fibrillation has been shown to be non-inferior to early cardioversion [7]. In addition, literature reports conflicting data regarding successful or failed ECV during blanking period and its effect on long-term rhythm outcome [8, 9]. Moreover, data of possible predictors for ECV outcome (successful or failed) for ERs are limited.

Hence, the purposes of the study were to investigate predictors for ECV outcome for the treatment of ERs post pulmonary vein isolation (PVI) and to study the association between timing and success of ECV after PVI on long-term rhythm outcome.

## Methods

### Study population

All patients at the Karolinska University Hospital between January 2012 and December 2017 were enrolled provided that catheter ablation for atrial fibrillation was a first-time pulmonary vein isolation (PVI) procedure using radiofrequency (RF) technique. Patients subjected to additional ablation lines in the right/left atrium or ablation of complex fractionated atrial electrograms were excluded. Likewise, patients experiencing periprocedural major complications such as cardiac tamponade, cerebrovascular events, major bleeding, and AV fistula were excluded. Requirement for inclusion was that patients presented with ER post ablation which was treated by ECV during blanking period. Type of atrial fibrillation (paroxysmal or persistent) was defined according to the current European guidelines [10]. Complete follow-up information had to be available for 12 months post-PVI. Relevant patient characteristics and procedural details were prospectively collected at the time of the ablation procedure and recorded in a computerized database. All patient data and follow-up information were derived from the digital medical record system (TakeCare, CompuGroup Medical Sweden, Uppsala, Sweden) which covers most of the hospitals and medical practices in Stockholm County.

### Catheter ablation procedure

Oral anticoagulation therapy was prescribed at least 1 month before the procedure. Transesophageal echocardiography prior to the procedure was performed to exclude left atrial appendage thrombus in all patients. The ablation procedures were performed under conscious sedation and analgesia. Throughout the procedure, a continuous infusion of heparin was maintained to achieve an activated clotting time (ACT) of > 300 s and ACT measurements were routinely done every 30 min. All patients underwent sole circumferential PVI as described before at our institution [11]. In brief, vascular access was obtained using the right and/or left femoral vein. Under fluoroscopic guidance trans-septal access to the left atrium was established through which the RF ablation catheter (Biosense Webster Inc., Diamond Bar, CA, USA) and circular mapping catheter (Lasso, Biosense Webster Inc., Diamond Bar, CA, USA) guided by a 3-dimensional mapping system (Carto, Biosense Webster Inc., Diamond Bar, CA, USA, or NavX, St. Jude Medical Inc., St. Paul, MN, USA) were advanced into the left atrium. Circumferential lesions were created to surround the right and left pulmonary veins (PV) with a 3.5-mm irrigated-tip catheter (Biosense Webster Inc., Diamond Bar, CA, USA). RF energy was applied with a power between 25 and 35 W, with an irrigation rate of 10 to 40 mL/min.

Acute procedural success was defined as entrance and exit block at least 20 min after initial PVI, documented with a circular mapping catheter. Pulmonary veins (PVs) with acute reconnection were re-isolated. Application of adenosine to assess for dormant PV conduction after ablation was not performed. All patients who underwent ablation were treated with the same approach.

### Post-ablation follow-up

Post ablation, patients were closely monitored for post-procedural complications and discharged home after 24 h. Oral anticoagulation was continued for at least 3 months post ablation. Further use of oral anticoagulation was determined according to the ESC guidelines [10]. At the discretion of the treating physician, prescription of antiarrhythmic drugs (AADs; i.e., amiodarone, dronedarone, disopyramid, flecainid, or sotalol) during the 3-month blanking period was performed to favor reverse electrical and structural atrial remodelling. AADs were stopped in all symptom-free patients not later than the end of the blanking period.

Patients were followed up for 1 year with clinical visits in the outpatient clinic and at medical practices scheduled at 3, 6, and 12 months post procedure. An ambulatory ECG and/or 24-h Holter ECG (at least one Holter ECG during follow-up) was routinely obtained during follow-up visits as well as during unscheduled ambulatory visits related to arrhythmia recurrences. For patients with an implantable cardiac device, the device was interrogated for arrhythmia burden at each clinic visit. Documentation of arrhythmic episodes was based on ECG, Holter ECG, or implanted device recordings (when available).

Recurrences were defined as any documented atrial tachyarrhythmias (atrial fibrillation, atrial flutter, atrial tachycardia) lasting > 30 s. In the case of symptomatic episodes, onset of recurrence was defined as timepoint of first sensation of atrial tachyarrhythmia experienced by the patient followed by a documentation of atrial tachyarrhythmia. In the case of asymptomatic atrial tachyarrhythmia, timepoint of documentation in the ECG was counted as recurrence onset. Early recurrence (ER) was defined as any atrial tachyarrhythmia occurring during the first 3 months post ablation. Late recurrence (LR) was defined as any atrial tachyarrhythmia occurring after the 3 months blanking period (first episode of recurrence was documented).

### Electrical cardioversion

Restoration of SR was aimed for in all patients with ER and ECV was indicated at the discretion of the treating physician and according to guidelines [12]. Anteroposterior transthoracic synchronized ECV applying an external defibrillator (Heart Start, Philips) was performed under sedation with intravenous propofol with biphasic shock energy applying 200 J up to 3 times until restoration of sinus rhythm. Successful ECV was defined as termination of atrial fibrillation and restoration of sinus rhythm after shock delivery. Failure of ECV was defined as relapse of atrial fibrillation within 5 min after shock delivery. In the case of several ECVs during blanking period, ECV was counted as failure if at least one failed ECV was present even if ECV was successful at another timepoint. In order to investigate timing of ECV related to the onset of ER, performance of ECV was divided into ≤ 7 days vs > 7 days from ER onset.

## Statistical analysis

All continuous variables are presented as mean ± standard deviation or median and interquartile range and were compared by using Student’s *t* tests or Mann–Whitney tests, respectively. Categorical variables are expressed as frequencies/percentages and were compared by chi-square tests. Univariate and multivariable backward logistic regression analyses were performed to identify factors associated with ECV failure and LR. The multivariable model considered factors associated with a *p*-value < 0.1 in univariate analyses and removed variables with *p* < 0.1 in a stepwise approach. The Kaplan–Meier method was used for building event curves. Hazard ratios with 95% confidence intervals and *p*-values from the Cox regression and Log-rank analyses are provided. All statistical tests and confidence intervals were 2-sided, with a significance level of 0.05. Statistical analyses were performed using SPSS software, version 25 (IBM Corp., Armonk, NY).

## Results

### Baseline characteristics

**Fig. 1:**
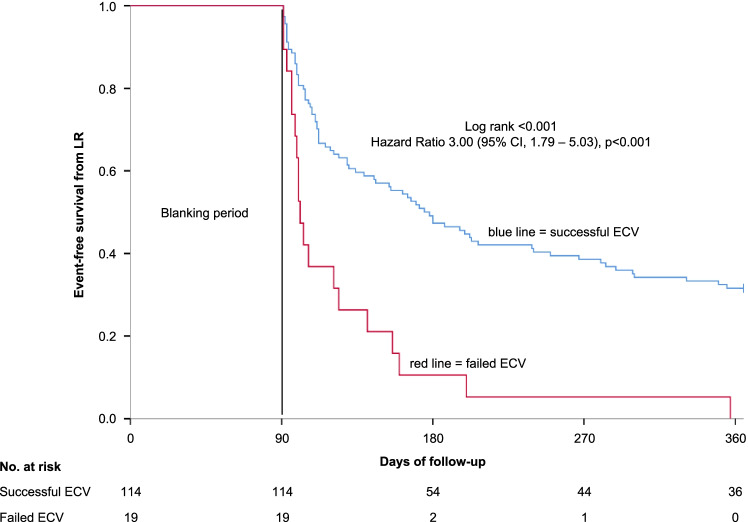
Kaplan–Meier analysis of event-free survival from LR in patients with successful ECV compared to failed ECV during blanking period after a 1-year follow-up. LR, late recurrence; ECV, electrical cardioversion. Blanking period = first 90 days post ablation

Of the 1836 patients who underwent ablation for atrial fibrillation, 713 fulfilled inclusion criteria. Of these, 337 experienced an ER during the blanking period (i.e., within 90 days post ablation) which was treated by ECV in 133 patients (Fig. 1, supplementary). These patients were divided into two groups depending on whether the ECV was successful (114 patients (85.7%)) or failed (19 patients (14.3%)). Patients with failed ECV had a significantly higher body mass index (29.6 kg/m^2^ vs 27.4 kg/m^2^, *p* = 0.009) and a lower left ventricular ejection fraction (52.4% vs 56.1%, *p* = 0.018), showed a higher proportion with AAD treatment during the blanking period (78.9% vs 50.9%, *p* = 0.026), and had a longer procedure time of ablation (209.1 min vs 180.3 min, *p* = 0.031) compared to patients with successful ECV. All baseline clinical characteristics of the study participants are presented in Table 1.

**Table 1:** Baseline characteristics of patients with early recurrence and electrical cardioversion (successful vs failed ECV) during blanking period

Baseline characteristics	All patients (*n* = 133)	Successful ECV (*n* = 114)	Failed ECV (*n* = 19)	*p*-value
Age (years)	60.9 ± 9.4	61.1 ± 8.8	59.4 ± 12.4	0.471
Male, *n* (%)	86 (64.7)	74 (64.9)	12 (63.2)	1.000
BMI (kg/m^2^)	27.7 ± 3.4	27.4 ± 3.0	29.6 ± 4.5	0.009
Type of atrial fibrillation				0.602
Paroxysmal, *n* (%)	42 (31.6)	35 (30.7)	7 (36.8)	
Persistent, *n* (%)	91 (68.4)	79 (69.3)	12 (63.2)	
Duration of atrial fibrillation in the past, years*	5.0 (2.0; 8.0)	5.0 (2.0; 8.0)	4.0 (2.0; 6.0)	0.210
Number of failed AADs	1.2 ± 0.7	1.2 ± 0.1	1.3 ± 0.1	0.431
Previous ECV, *n* (%)	105 (78.9)	92 (80.7)	13 (68.4)	0.233
CHA_2_DS_2_-VASc score, *n* (%)				0.936
0	23 (17.3)	20 (17.5)	3 (15.8)	
1	45 (33.8)	37 (32.5)	8 (42.1)	
2	38 (28.6)	33 (28.9)	5 (26.3)	
≥ 3	27 (20.3)	24 (21.1)	3 (15.8)	
Arterial hypertension, *n* (%)	71 (53.4)	62 (54.4)	9 (47.4)	0.625
Diabetes mellitus, *n* (%)	6 (4.5)	4 (3.5)	2 (10.5)	0.204
Hyperlipidemia, *n* (%)	26 (19.5)	24 (21.1)	2 (10.5)	0.365
Smoker, *n* (%)	7 (5.3)	6 (5.3)	1 (5.3)	1.000
Left atrial size, parasternal long axis (mm)	41.5 ± 4.2	41.4 ± 4.0	42.0 ± 5.1	0.548
Left ventricular ejection fraction (%)	55.6 ± 6.4	56.1 ± 6.1	52.4 ± 7.4	0.018
Beta-blocker at discharge, *n* (%)	118 (88.7)	102 (86.4)	16 (84.2)	0.450
AAD treatment during blanking period^#^, *n* (%)	73 (54.9)	58 (50.9)	15 (78.9)	0.026
Procedure time (min)	184.4 ± 54.0	180.3 ± 49.6	209.1 ± 72.1	0.031
Fluoroscopy time (min)	18.5 ± 17.7	18.2 ± 18.8	20.3 ± 8.9	0.629
Radiofrequency delivery time (s)	2753.7 ± 1020.9	2734.6 ± 984.7	2872.4 ± 1249.0	0.597
ECV ≤ 7 days from ER onset	88 (66.2)	79 (69.3)	9 (47.4)	0.071
Timepoint of ECV during blanking period^#^, *n* (%)				0.907
During 1st month	77 (57.9)	66 (57.9)	11 (57.9)	
During 2nd month	32 (24.1)	28 (24.6)	4 (12.5)	
During 3rd month	24 (18.0)	20 (17.5)	4 (21.1)	

^#^Blanking period = first 90 days post ablation

^*^Non-normally distributed continuous variables are expressed as median and interquartile range (25th and 75th percentile)

*ER*, early recurrence; *ECV*, electrical cardioversion; *BMI*, body mass index; *AAD*, antiarrhythmic drug. AADs include amiodarone, dronedarone, disopyramid, flecainid, and sotalol

### Associations with ECV failure

Of the 19 patients with ECV failure, 15 patients (78.9%) had transient restoration of sinus rhythm and immediate reinitiation of atrial fibrillation within 5 min and 4 patients (21.1%) never converted to sinus rhythm during ECV failure. Nine patients (47.4%) received at least one additional ECV and 10 patients (52.6%) received additional AAD treatment after ECV failure during blanking period.

Table 2 presents the results of univariate and multivariable regression analyses of associations with ECV failure. In multivariable analysis, a higher body mass index (odds ratio (OR) 1.19 (95% CI 1.02–1.39), *p* = 0.029), a lower left ventricular ejection fraction (OR 1.07 (95% CI 0.99–1.15), *p* = 0.079), AAD treatment during blanking period (OR 4.22 (95% CI 1.23–14.52), *p* = 0.022), and performance of ECV > 7 days from ER onset (OR 2.99 (95% CI 1.01–8.87), *p* = 0.048) were independently associated with ECV failure (Table 2).

**Table 2:** Univariate and multivariable regression analyses for ECV failure

Variable	Univariate analysis		Multivariable analysis	
	Odds ratio (95% CI)	*p*-value	Odds ratio (95% CI)	*p*-value
BMI	1.21 (1.04–1.40)	0.012	1.19 (1.02–1.39)	0.029
Left ventricular ejection fraction	1.08 (1.01–1.15)	0.025	1.07 (0.99–1.15)	0.079
AAD treatment during blanking period^#^	3.62 (1.13–11.58)	0.030	4.22 (1.23–14.52)	0.022
ECV > 7 days from ER onset	2.51 (0.94–6.71)	0.067	2.99 (1.01–8.87)	0.048

*CI*, confidence interval; *BMI*, body mass index; *ECV*, electrical cardioversion; *AAD*, antiarrhythmic drug

^#^Blanking period = first 90 days post ablation

### Late recurrence

Of the total study group, 97 patients (72.9%) developed LRs. During 1-year follow-up, the rate of LR was significantly higher among patients with failed ECV as compared to patients with successful ECV (LR rate 100% vs 68.4%; hazard ratio (HR) 3.00 (95% CI, 1.79–5.03), *p* < 0.001). The corresponding Kaplan–Meier curve is provided in Fig. 1. Notably, in this cohort, all patients with a failed ECV developed LRs although in 15 patients (78.9%) sinus rhythm was transiently restored during blanking period after ECV failure.

**Fig. 2:**
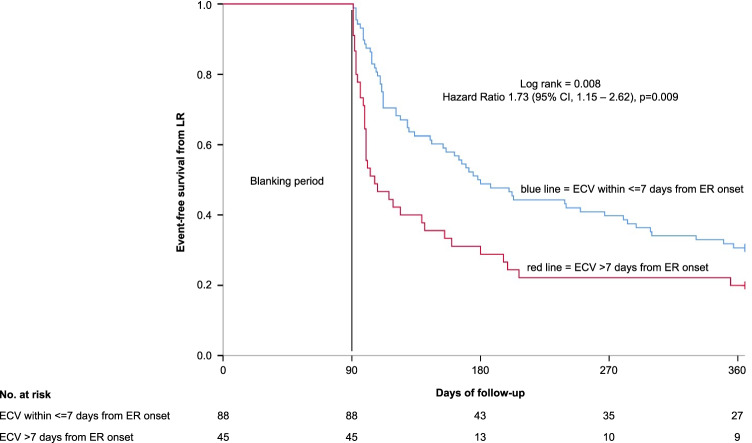
Kaplan–Meier analysis of event-free survival from LR in patients with ECV within ≤ 7 days from ER onset compared to patients with ECV > 7 days from ER onset during blanking period after a 1-year follow-up. LR, late recurrence; ECV, electrical cardioversion; ER, early recurrence. Blanking period = first 90 days post ablation

In order to investigate whether timing of ECV related to the onset of ER has an impact on long-term rhythm outcome, we analyzed patients with ECV performed within ≤ 7 days from ER onset (88 patients (66.2%)) compared to patients with ECV performed > 7 days from ER onset (45 patients (33.8%)). Baseline characteristics of the patients did not significantly differ between the two groups (Table 1, supplementary). Patients with ECV performed > 7 days from ER onset had a significantly higher risk of developing LR as compared to patients with ECV performed within ≤ 7 days from ER onset (LR rate 80% vs 69.3%; HR 1.73 (95% CI 1.15–2.62), *p* = 0.009). The corresponding Kaplan–Meier curve is provided in Fig. 2.

### Associations with LR

Table 3 presents the results of univariate and multivariable regression analyses of associations with LR. In multivariable analysis, persistent atrial fibrillation (HR 1.61 (95% CI 1.02–2.52), *p* = 0.039), longer duration of atrial fibrillation in the past (HR 1.003 (95% CI 1.000–1.006), *p* = 0.025), performance of ECV > 7 days from ER onset (HR 1.76 (95% CI 1.16–2.67), *p* = 0.008), and failed ECV (HR 3.32 (95% CI 1.96–5.64), *p* < 0.001) were independently associated with LR (Table 3).

**Table 3:** Univariate and multivariable regression analyses for LR

Variable	Univariate analysis		Multivariable analysis	
	Hazard ratio (95% CI)	*p*-value	Hazard ratio (95% CI)	*p*-value
Persistent atrial fibrillation	1.49 (0.95–2.32)	0.080	1.61 (1.02 – 2.52)	0.039
Duration of atrial fibrillation in the past	1.002 (1.000–1.005)	0.092	1.003 (1.000–1.006)	0.025
ECV > 7 days from ER onset	1.73 (1.15–2.62)	0.009	1.76 (1.16–2.67)	0.008
Failed ECV	3.00 (1.79–5.03)	< 0.001	3.32 (1.96–5.64)	< 0.001

*LR*, late recurrence; *CI*, confidence interval; *BMI*, body mass index; *ECV*, electrical cardioversion; *AAD*, antiarrhythmic-drug

^#^ Blanking period = first 90 days post ablation

### Consistency analysis

In order to investigate whether the observed results were also found in different subgroups stratified according to their type of atrial fibrillation (paroxysmal atrial fibrillation (*n* = 42) vs persistent atrial fibrillation (*n* = 91)), we performed a consistency analysis only considering these two distinct groups.

In patients with paroxysmal atrial fibrillation, the rate of LR was significantly higher among patients with failed ECV (*n* = 7 (16.7%)) as compared to patients with successful ECV (*n* = 35 (83.3%)) (HR 4.88 (95% CI, 1.81–13.15), *p* = 0.002) during 1-year follow-up. Patients with ECV performed > 7 days from ER onset (*n* = 15 (35.7%)) had a significantly higher risk of developing LR as compared to patients with ECV performed within ≤ 7 days from ER onset (*n* = 27 (64.3%)) (HR 2.20 (95% CI 1.03–4.72), *p* = 0.049).

In patients with persistent atrial fibrillation, the rate of LR was significantly higher among patients with failed ECV (*n* = 12 (13.2%)) as compared to patients with successful ECV (*n* = 79 (86.8%)) (HR 2.85 (95% CI, 1.52–5.35), *p* = 0.001) during 1-year follow-up. Patients with ECV performed > 7 days from ER onset (*n* = 30 (33.0%)) had a higher risk of developing LR as compared to patients with ECV performed within ≤ 7 days from ER onset (*n* = 61 (67.0%)) (HR 1.636 (95% CI 0.996–2.686), *p* = 0.052). Hence, the results from consistency analysis were in line with the primary analysis of the whole study cohort.

## Discussion

In this study, we identified several predictors for ECV failure such as a higher body mass index, a lower left ventricular ejection fraction, and performance of ECV > 7 days from ER onset. Failed ECV and performance of ECV > 7 days from ER onset remained independently associated with LR.

A failed ECV during the first 90 days after PVI went along with a 100% LR rate and remained independently associated with LR in our cohort. This is in line with the study of Nakamaru et al. [9] where a failed ECV for ER was an independent predictor of LR. In another study, ECV failure was not associated with long-term rhythm outcome [8]. In the latter study, only ERs within the first 7 days post ablation were studied which might generate the difference of results.

Since the outcome of ECV is a crucial factor for long-term rhythm outcome, it is important to identify predictors for ECV success/failure. The knowledge of potentially modifiable risk factors and comorbidities gives the possibility that a treatment priority of those may facilitate maintenance of sinus rhythm after ECV [13]. In former studies associated with the general ECV treatment of atrial fibrillation, several risk factors such as diabetes, renal impairment, or arterial hypertension have been identified [14–16]. A lower body weight < 80 kg has been shown to be an independent predictor for the success of ECV [17] since patients who have a higher body weight may have greater energy requirements for successful ECV. The presence of LV dysfunction has been shown to have a negative impact upon ECV success [15]. In our study, we identified that a higher body mass index, a lower left ventricular ejection fraction, and a late ECV (> 7 days from ER) are risk factors for ECV failure. This goes in line with former studies and shows that these factors also play a pivotal role in the ECV treatment of ER.

In our study, an early ECV, i.e., within 7 days from ER onset, went along with a better long-term rhythm outcome. This is in accordance with the study of Malasana et al. [6] which showed that an aggressive strategy with rapid ECV within 24 h from ER onset was beneficial in terms of long-term rhythm outcome. In addition, in the study of Baman et al. [18], an early ECV within 30 days from ER onset was the only independent predictor of maintenance of sinus rhythm after a single ablation procedure. The early ECV strategy for ER is suggested to avoid that atrial fibrillation itself may adversely affect the atrial myocardium on a cellular and inflammatory level abolishing the favorable effects of the ablation.

Previous studies did not provide consistent data regarding the treatment of ERs with ECV and its impact on long-term rhythm outcome. Currently, it is recommended to cardiovert patients with persistent atrial arrhythmia post atrial fibrillation ablation preferably within 30 days of arrhythmia onset [1]. However, the clinical data available supporting this approach remain limited. Therefore, the knowledge of factors associated with a beneficial effect of ECV on long-term rhythm outcome is of relevance. Integration of relevant factors in a scoring system as done for general ECV treatment of acute atrial fibrillation [19] may even help to better select patients that benefit from an ECV treatment approach for ER.

In our study, performance of ECV > 7 days from ER onset remained independently associated with LR. Risk factors such as a higher body mass index and a lower left ventricular ejection fraction contributing to a failed ECV outcome should be avoided or modified, when possible, to facilitate maintenance of sinus rhythm after ECV.

## Limitations

This is a cohort study of registry-based design. All data were collected at a single electrophysiology center and, therefore, results may differ from other centers. It is possible that the exact onset of asymptomatic atrial tachyarrhythmias or entire asymptomatic episodes may have been missed since follow-up post ablation did not include intensive monitoring by, e.g., transtelephonic monitoring or implantable loop recorder. All ablation procedures were performed only applying PVI using RF technique. Hence, our results may not be applicable to other forms of energy delivery such as cryoablation as well as more complex ablation techniques. The latter especially accounts for patients with persistent atrial fibrillation potentially having more complex PV substrates predisposing for LR. ECVs were performed according to clinical routine and were not standardized which could potentially introduce a bias into the results.

## Conclusion

In this study, a failed ECV within 90 days for ER after PVI for paroxysmal and persistent atrial fibrillation and performance of ECV > 7 days from ER onset were independently associated with LR and, thus, are potential early indicators for the need of a redo ablation procedure. Our study provides valuable insights into the management of ERs helping to characterize better the mechanisms of atrial fibrillation and its long-term outcome. Large-scale prospective randomized studies are warranted to understand which subset of patients benefits from ECV for ER and at what timepoint the ECV should be performed.

## Supplementary Information

Below is the link to the electronic supplementary material.

Supplementary file1 (PDF 21 KB) Figure 1 PRISMA flow diagram showing the selection process for all patients

Supplementary file2 (DOCX 18 KB)

## Data Availability

The data underlying this article cannot be shared publicly due to privacy of individuals that were investigated in the study. The data will be shared on reasonable request to the corresponding author provided that this in accordance with the institutional ethical guidelines as well as regulation and legislation.
