# Supplementary material for: Electrical cardioversion for early recurrences post pulmonary vein isolation
Source: J Interv Card Electrophysiol. 2022 Sep 9;66(3):577–84. doi: 10.1007/s10840-022-01368-w (PMC10066117; doi:10.1007/s10840-022-01368-w)
Supplement: Supplementary file 1 — Supplementary file1 (PDF 21 KB) Figure 1 PRISMA flow diagram showing the selection process for all patients [file 10840_2022_1368_MOESM1_ESM.pdf]

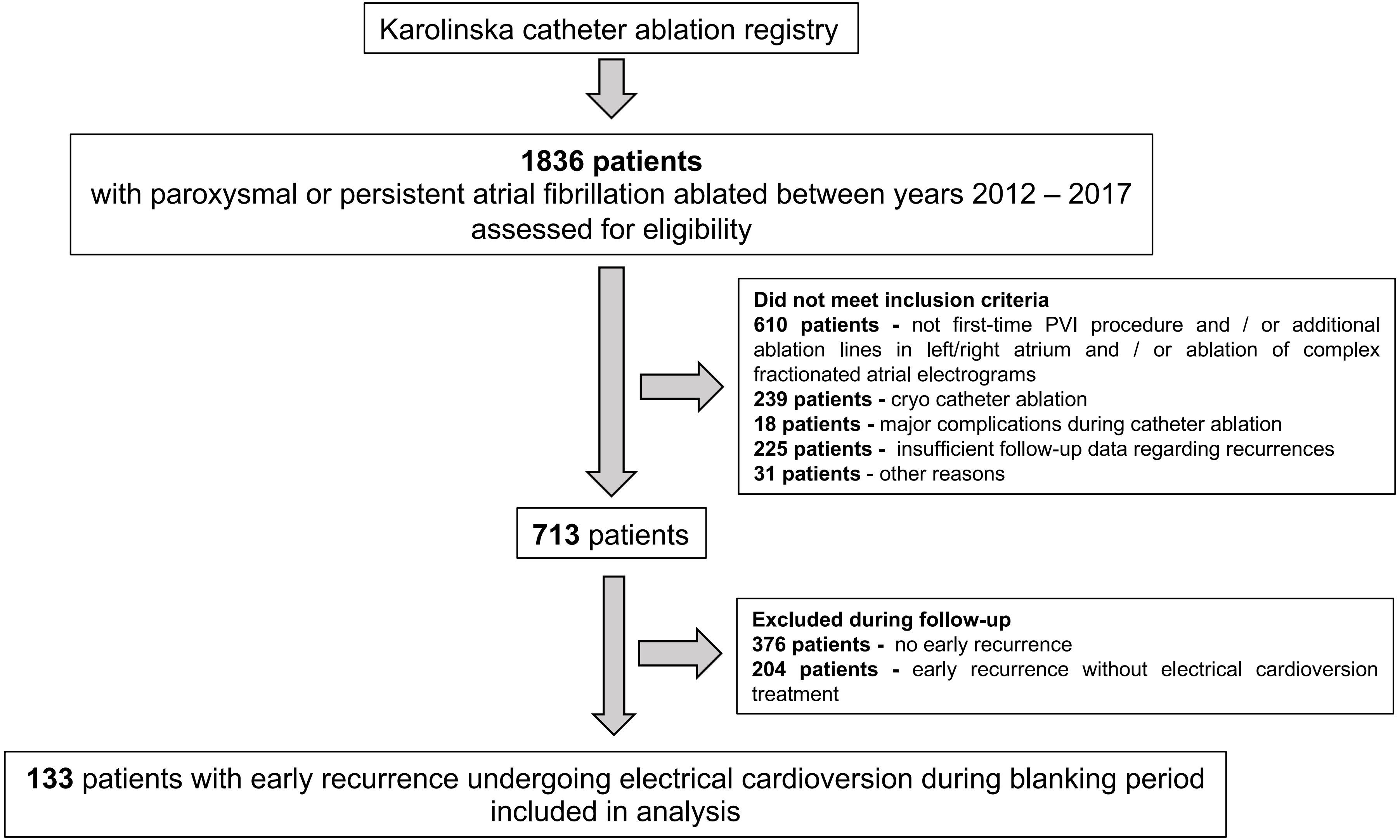
Karolinska catheter ablation registry
1836 patients with paroxysmal or persistent atrial fibrillation ablated between years 2012 – 2017 assessed for eligibility
Did not meet inclusion criteria
610 patients - not first-time PVI procedure and / or additional ablation lines in left/right atrium and / or ablation of complex fractionated atrial electrograms
239 patients - cryo catheter ablation
18 patients - major complications during catheter ablation
225 patients - insufficient follow-up data regarding recurrences
31 patients - other reasons
713 patients
Excluded during follow-up
376 patients - no early recurrence
204 patients - early recurrence without electrical cardioversion treatment
133 patients with early recurrence undergoing electrical cardioversion during blanking period included in analysis
