# Supplementary material for: Electrical cardioversion for early recurrences post pulmonary vein isolation
Source: J Interv Card Electrophysiol. 2022 Sep 9;66(3):577–84. doi: 10.1007/s10840-022-01368-w (PMC10066117; doi:10.1007/s10840-022-01368-w)
Supplement: Supplementary file 2 — Supplementary file2 (DOCX 18 KB) [file 10840_2022_1368_MOESM2_ESM.docx]

**Supplementary**

Table 1

Baseline characteristics of patients with ER treated by ECV within <= 7 days vs >7days from ER onset.

| **Baseline characteristics** | **ECV <= 7 days from ER onset (n=88)** | **ECV > 7days from ER onset (n=45)** | **p-value** |
| --- | --- | --- | --- |
| Age (years) | 61.3 +/- 8.8 | 60.0 +/- 10.4 | 0.449 |
| Male, n (%) | 56 (63.6) | 30 (66.7) | 0.848 |
| BMI (kg/m^2^) | 27.8 +/- 3.3 | 27.6 +/- 3.6 | 0.687 |
| Type of atrial fibrillation |  |  | 0.844 |
| Paroxysmal, n (%) | 27 (30.7) | 15 (33.3) |  |
| Persistent, n (%) | 61 (69.3) | 30 (66.7) |  |
| Duration of atrial fibrillation in the past, years* | 5.0 (3.0; 8.0) | 5.0 (1.5; 8.5) | 0.229 |
| Number of failed AADs | 1.2 +/- 0.8 | 1.2 +/- 0.8 | 0.727 |
| Previous ECV, n (%) | 70 (79.5) | 35 (77.8) | 0.825 |
| CHA₂DS₂-VASc Score, n (%) |  |  | 0.609 |
| 0 | 14 (15.9.) | 9 (20.0) |  |
| 1 | 33 (37.5) | 12 (26.7) |  |
| 2 | 22 (25.0) | 16 (35.6) |  |
| >=3 | 19 (21.6) | 8 (17.8) |  |
| Arterial Hypertension, n (%) | 48 (54.5) | 23 (51.1) | 0.718 |
| Diabetes mellitus, n (%) | 4 (4.5) | 2 (4.4) | 1.000 |
| Hyperlipidemia, n (%) | 20 (22.7) | 6 (13.3) | 0.251 |
| Smoker, n (%) | 5 (5.7) | 2 (4.4) | 1.000 |
| Left atrial size, parasternal long axis (mm) | 41.3 +/- 4.3 | 41.7 +/- 4.0 | 0.609 |
| Left ventricular ejection fraction (%) | 56.0 +/- 6.6 | 54.8 +/- 6.0 | 0.310 |
| Beta-blocker at discharge, n (%) | 80 (90.9) | 38 (84.4) | 0.385 |
| AAD treatment during blanking period^#^, n (%) | 50 (56.8) | 23 (51.1) | 0.583 |
| Procedure time (min) | 186.8 +/- 54.2 | 179.8 +/- 54.1 | 0.487 |
| Fluoroscopy time (min) | 19.2 +/- 20.9 | 17.0 +/- 8.7 | 0.519 |
| Radiofrequency delivery time (sec) | 2849.9 +/- 987.6 | 2571.8 +/- 1068.6 | 0.140 |

^#^ blanking period = first 90 days post ablation

*Non-normally distributed continuous variables are expressed as median and interquartile range (25^th^ and 75^th^ percentile).

ER = early recurrence, ECV = electrical cardioversion, BMI = body-mass-index, AAD = antiarrhythmic-drug; AADs include: Amiodarone, Dronedarone, Disopyramid, Flecainid, Sotalol
